# Transfer of Ho Endonuclease and Ufo1 to the Proteasome by the UbL-UbA Shuttle Protein, Ddi1, Analysed by Complex Formation *In Vitro*

**DOI:** 10.1371/journal.pone.0039210

**Published:** 2012-07-16

**Authors:** Olga Voloshin, Anya Bakhrat, Sharon Herrmann, Dina Raveh

**Affiliations:** Department of Life Sciences, Ben Gurion University of the Negev, Beersheba, Israel; University of Pittsburgh, United States of America

## Abstract

The F-box protein, Ufo1, recruits Ho endonuclease to the SCF^Ufo1^ complex for ubiquitylation. Both ubiquitylated Ho and Ufo1 are transferred by the UbL-UbA protein, Ddi1, to the 19S Regulatory Particle (RP) of the proteasome for degradation. The Ddi1-UbL domain binds Rpn1 of the 19S RP, the Ddi1-UbA domain binds ubiquitin chains on the degradation substrate. Here we used complex reconstitution *in vitro* to identify stages in the transfer of Ho and Ufo1 from the SCF^Ufo1^ complex to the proteasome. We report SCF^Ufo1^ complex at the proteasome formed in the presence of Ho. Subsequently Ddi1 is recruited to this complex by interaction between the Ddi1-UbL domain and Ufo1. The core of Ddi1 binds both Ufo1 and Rpn1; this interaction confers specificity of SCF^Ufo1^ for Ddi1. The substrate-shield model predicts that Ho would protect Ufo1 from degradation and we find that Ddi1 binds Ho, Ufo1, and Rpn1 simultaneously forming a complex for transfer of Ho to the 19S RP. In contrast, in the absence of Ho, Rpn1 displaces Ufo1 from Ddi1 indicating a higher affinity of the Ddi1-UbL for the 19S RP. However, at high Rpn1 levels there is synergistic binding of Ufo1 to Ddi1 that is dependent on the Ddi1-UbA domain. Our interpretation is that in the absence of substrate, the Ddi1-UbL binds Rpn1 while the Ddi1-UbA binds ubiquitin chains on Ufo1. This would promote degradation of Ufo1 and disassembly of SCF^Ufo1^ complexes.

## Introduction

The Ubiquitin-proteasome system has a major role in regulation of cellular processes, in particular the cell cycle and many signaling pathways [1], [2]. Proteins targeted for degradation are conjugated to ubiquitin (Ub) by a cascade of enzymes, an E1 Ub activating- and E2 Ub conjugating enzyme, and an E3 Ub ligase responsible for substrate identification [3]. In some instances an E4 Ub chain elongating activity is also involved [4]. Ub chains comprising at least four K48-linked Ub molecules are recognized by the 19S Regulatory particle (RP) of the proteasome, either by an endogenous 19S RP subunit [5]–[7], or by a member of the UbL-UbA protein family. UbL-UbA proteins bind specific 19S RP subunits through their Ub-like (UbL) domain and K48-Ub chains on the substrate through their Ub-associated (UbA) domain. The yeast family of UbL-UbA proteins comprises Rad23, Dsk2, and Ddi1, and each family member participates in the degradation of a range of substrates either by itself, or as a Rad23-Dsk2 pair (reviewed in [8]).

UbL-UbA proteins are often referred to as shuttle proteins based on their recruitment of the ubiquitylated substrate from the E2-E3 complex and transfer to the 19S RP. This is supported particularly by the interaction between Rad23 and Dsk2 with the chain elongating E4, Ufd2, that occurs in the framework of a complex between Ufd2 and the AAA-ATPase ring hexamer, Cdc48 [9]. However, many E3s bind the 19S RP directly: these include Ubr1 and Ufd4 [10], Hul5 [11], Ufo1 [12], SCF (Skp1-Cullin1-F-box protein) and APC (Anaphase Promoting complex) [13], [14]. In the case of Ufd4, direct interaction between the E3 and the proteasome is essential for substrate degradation [15]. In some instances the UbL-UbA protein may be an essential stochiometric subunit of the E3 complex, as reported for KPC2 (Kip1 ubiquitylation-promoting complex 2) that regulates degradation of the p27 cell cycle inhibitor [16]. These reports raise the question whether other UbL-UbA proteins may also occur as intrinsic components of an E3-19S RP complex and if so whether it is possible to detect additional interactions between the core domain of the UbL-UbA protein and subunits of this complex. In the event of such interactions are they a prerequisite for interaction of the E3 complex with the 19S RP?

The SCF complex comprises a rigid cullin scaffold, in *S. cerevisiae* Cdc53, with the RING protein, Rbx1, attached to a C-terminal domain [17]. The RING domain serves as a landing pad for the Ub-charged E2, Cdc34 [18]. Substrate recruitment is executed by a series of different F-box proteins (FBPs), each of which binds a subset of targets many of which are recognized by phosphorylation [19]–[21]. FBPs have a F-box domain and a WD40- or LRR substrate-binding domain. The F-box domain binds the Skp1 adaptor that interacts with the N-terminal domain of Cdc53 [17], [22]–[25]. Exchange of FBPs within the SCF complex is achieved by auto-ubiquitylation of the FBP followed by degradation in the proteasome [26], [27]. A number of FBPs of SCF complexes and the related BTB/3-box domain receptor proteins [28] have been shown to occur as homo- or heterodimers. These include homodimers of yeast Cdc4 and of human Fbw7 FBP [29] and the heterodimeric *S. pombe* Pop1-Pop2 FBPs [30], [31]. FBP dimerization was shown to be required for dimerization of Cdc53 [32] and increasing experimental data support a model of a dimeric cullin-RING ligase complex. Indeed although monomeric FBPs bind their substrates and Skp1, substrate ubiquitylation was reported to require their dimerization [28], [32], [33].

Ddi1 is required for the final stages of proteasomal degradation of both Ho endonuclease [34] and of Ufo1, its cognate FBP [35]. Ubiquitylated Ho interacts with the UbA domain of Ddi1 via its ubiquitin chains and its transfer to the 19S RP requires the UbL domain of Ddi1 that interacts with the LRR domain of the 19S RP subunit, Rpn1 [36]. Ddi1 forms a homodimer mediated by residues in its core (residues 180–325) giving rise to an active aspartyl protease site [37], [38]. In *ddi1*Δ mutants ubiquitylated Ho endonuclease accumulates in the cytoplasm and is not transferred to the proteasome for degradation [34]. Ufo1 and its fungal orthologs are unique FBPs as they have four copies of the Ub interacting motif (UIMs) at their C-terminus in addition to the F-box and WD40 domains present in other FBPs [35], [39], [40]. The UIM is a simple α-helical ubiquitin binding domain [41] and the Ufo1-UIMs are separated by long linkers suggesting this is a flexible region. Turnover of Ufo1 is dependent on an interaction between its UIMs and the UbL domain of Ddi1 [35]. Furthermore the *rpn1-D517A* mutation that disrupts binding of Ddi1 to the proteasome stabilizes Ufo1 [36].

A protein fragment comprising the Ufo1-UIMs interacts with all three UbL-UbA proteins, Rad23, Dsk2, and Ddi1, however full-length (FL) Ufo1 interacts only with Ddi1 suggesting that the core residues are important for specificity [35]. UIMs have been shown to interact with Ub-charged E2s to promote monoubiquitylation of a different domain of their host protein [42]–[45]. Deletion of *UFO1* has no obvious phenotype under normal growth conditions, however, a genomic *UFO1* allele deleted for the UIMs is dominant lethal. Ectopic high level expression of *UFO1* without the UIMs leads to stabilization of the protein and to cell cycle arrest at the end of G_1_. Substrates of other FBPs accumulate suggesting that Ddi1 is required for disassembly of SCF^Ufo1^ complexes and recycling of the core complex subunits into alternative SCF complexes [35].

Here we used complex reconstitution *in vitro* to augment our *in vivo* data showing a role for Ddi1 in degradation of Ho and of its cognate FBP, Ufo1 [34], [35], [40], [46]. In particular we aimed to identify stages in the handover of Ho from the SCF^Ufo1^ complex to the 19S RP and subsequent degradation of Ufo1. We delineate stages in the formation of SCF^Ufo1^-Ho-Ddi1-19S RP complex. Domain analysis showing different modes of interaction of Ddi1 with Ufo1 and Rpn1 in the presence and absence of Ho support the “Substrate shield” model of protein degradation [47]. We present a model for sequential handover of Ho and Ufo1 to the proteasome.

## Results

### Ufo1 Forms Dimers Initiated by the UIMs

**Figure 1:**
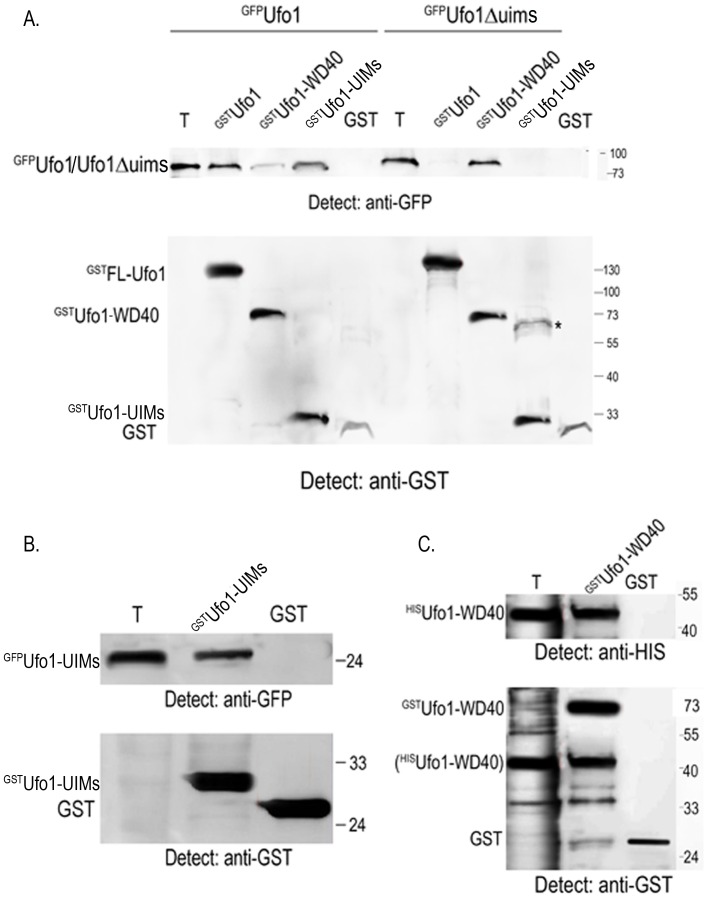
Ufo1 forms a homodimer via its UIMs. A.^ GST^FL-Ufo1, ^GST^Ufo1-WD40 domain, ^GST^Ufo1-UIMs or GST beads were incubated with yeast extract from cells expressing full-length p*GAL-GFP-UFO1* or p*GAL-GFP-UFO1Δuims.* The bead fraction was analysed by Western blotting with anti-GFP and anti-GST antibodies. T is 10% of yeast extract with which the beads were incubated. *denotes contaminant band. B. Recombinant ^GST^Ufo1-UIMs or control GST beads were incubated with yeast extract with ^GFP^Ufo1-UIMs and analysed as above. T is 10% of yeast extract as above. C. Recombinant ^GST^Ufo1 WD40 domain protein or control GST on GSH beads were incubated with bacterial lysate from cells that expressed^ HIS^Ufo1-WD40 and the bead fraction was analysed by Western blotting initially with anti-HIS and then with anti-GST antibodies. T is 10% of yeast extract as above. The brackets around ^HIS^Ufo1-WD40 in the anti-GST Western blot indicate that these bands were observed after incubation with anti-HIS antibodies as shown in the upper part of the blot.

Given the importance of FBP dimerization for substrate ubiquitylation [28], [32], [33] we examined whether Ufo1 forms a dimer. Furthermore we aimed to determine which domain(s) of Ufo1 could have a role in dimerization. We incubated yeast extract from cells that produced full-length (FL), ^GFP^FL-Ufo1, or Ufo1 truncated for the C-terminal UIMs, ^GFP^Ufo1Δuims, with GSH beads bound to recombinant ^GST^FL-Ufo1, ^GST^Ufo1-WD40 domain, ^GST^Ufo1-UIMs, or control GST (Figure S1). We observed a robust interaction of ^GFP^FL-Ufo1 with ^GST^FL-Ufo1 and with ^GST^Ufo1-UIMs whereas the interaction between ^GFP^FL-Ufo1 and ^GST^Ufo1-WD40 domain was extremely weak. ^GFP^Ufo1Δuims did not interact with ^GST^FL-Ufo1 or with ^GST^Ufo1-UIMs. However, in contrast to ^GFP^FL-Ufo1, truncated ^GFP^Ufo1Δuims interacted robustly with ^GST^Ufo1-WD40 (Figure 1A).

These results suggest both a positive and a negative role for the Ufo1-UIMs in Ufo1 dimerization. The positive role is indicated by the ability of FL-Ufo1 to dimerize with both FL-Ufo1 and with the isolated Ufo1-UIM fragment, whereas the negative role is indicated by the absence of dimerization between FL-Ufo1 and the Ufo1-WD40 domain fragment. This may indicate that the Ufo1-UIMs regulate access to the WD40 domain. To test directly whether the Ufo1-UIMs dimerize we incubated yeast extract with ^GFP^Ufo1-UIMs with recombinant ^GST^Ufo1-UIMs on beads. We observed a robust interaction that was not found with the control GST beads indicating that isolated Ufo1-UIMs fragments dimerize (Figure 1B). The interaction between ^GFP^Ufo1Δuims and GSTUfo1-WD40 (Figure 1A) suggests that the Ufo1-WD40 domain by itself can dimerize. Indeed when we expressed the Ufo1-WD40 domain in bacteria with two different epitope tags we observed that ^GST^Ufo1-WD40 bound to ^HIS^Ufo1-WD40 (Figure 1C). Thus Ufo1 resembles other FBPs in forming dimers and both the unique Ufo1-UIMs and the Ufo1-WD40 domain participate in dimerization. Dimerization via the Ufo1-WD40 domains is supported by our previous finding of turnover of Ho in *ufo1Δ* mutants that produce plasmid-encoded Ufo1Δuims [35].

### SCF^Ufo1^ Complexes Interact with the 19S RP *in vitro* Only in the Presence of Substrate

**Figure 2:**
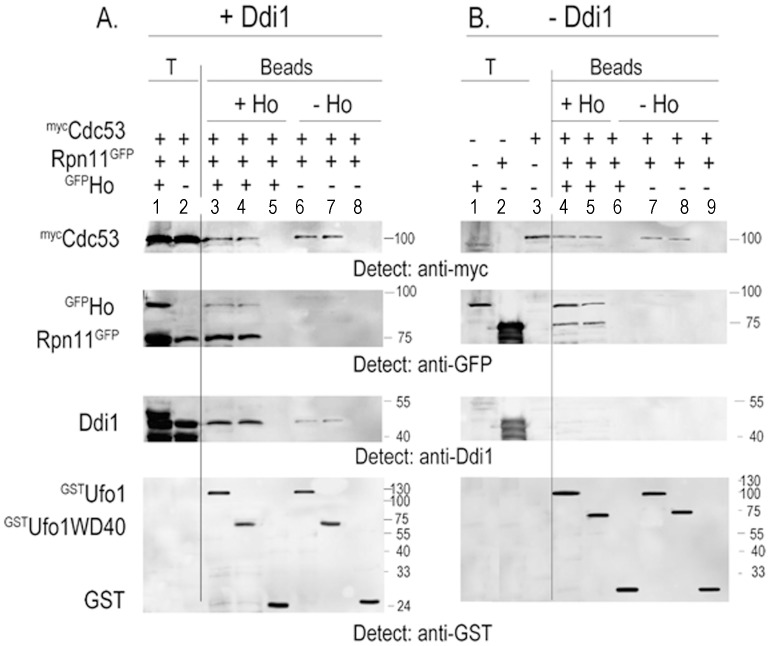
SCF^Ufo1^-Ho-19S RP complex formation *in vitro*. A. ^GST^Ufo1, ^GST^Ufo1-WD40 domain, or control GST beads were incubated with yeast extract from cells with tagged genomic *RPN11-GFP* that were transformed with p*GAL-MYC-CDC53* either with p*GAL*-*GFP-HO* or alone. The bead fraction was analysed by Western blotting with anti-GFP antibodies to detect Rpn11^GFP^ and ^GFP^Ho, with anti-myc antibodies to detect ^myc^Cdc53, and with anti-Ddi1 and anti-GST antibodies. T is 10% of total yeast extract with which the beads were incubated (Lanes 1 and 2). Lane 3: ^GST^Ufo1 beads incubated with ^myc^Cdc53, Rpn11^GFP^ and ^GFP^Ho; Lane 4: ^GST^Ufo1 WD40 domain incubated with ^myc^Cdc53, Rpn11^GFP^ and ^GFP^Ho; Lane 5: control GST beads incubated with these yeast extracts; Lane 6: ^GST^Ufo1 beads incubated with ^myc^Cdc53 and Rpn11^GFP^; Lane 7: ^GST^Ufo1 WD40 domain incubated with ^myc^Cdc53 and Rpn11^GFP^; Lane 8: control GST beads incubated with these yeast extracts. B. ^GST^Ufo1, ^GST^Ufo1-WD40, or control GST beads were incubated with yeast extract from *ddi1Δ* mutant cells that expressed p*GAL-MYC-CDC53,* p*GFP*-*RPN11,* with or without p*GAL*-*GFP-HO.* The bead fractions were analysed by Western blotting with anti-myc, anti-GFP, anti-Ddi1, and anti-GST antibodies as in A. T is 10% of total yeast extract with which the beads were incubated (Lanes 1-3). Lane 4: ^GST^Ufo1 beads incubated with ^myc^Cdc53, Rpn11^GFP^ and ^GFP^Ho; Lane 5: ^GST^Ufo1 WD40 domain incubated with ^myc^Cdc53, Rpn11^GFP^ and ^GFP^Ho; Lane 6: control GST beads incubated with these yeast extracts; Lane 7: ^GST^Ufo1 beads incubated with ^myc^Cdc53 and Rpn11^GFP^; Lane 8: ^GST^Ufo1 WD40 domain incubated with ^myc^Cdc53 and Rpn11^GFP^; Lane 9: control GST beads incubated with these yeast extracts.

Despite its nuclear role Ho must exit the nucleus to be degraded [46] and in *ddi1Δ* mutants stabilized Ho accumulates in the cytoplasm as an ubiquitylated conjugate [34]. SCF^Ufo1^ complexes that have bound Ho may associate with the 19S RP as reported for SCF^Cdc4^-Sic1 complexes [14], or alternatively Ddi1 could shuttle ubiquitylated Ho from a SCF^Ufo1^-Ho complex to the proteasome. We therefore reconstituted SCF^Ufo1^ complexes *in vitro* in the presence or absence of Ho. Recombinant ^GST^FL-Ufo1 and the ^GST^Ufo1-WD40 domain proteins on GSH beads were incubated with yeast extract from cells that produced ^myc^Cdc53 and with the 19S RP complex tagged with Rpn11^GFP^. The experiment was performed both in the presence and the absence of ^GFP^Ho endonuclease. Experimental conditions are such that the 19S RP complex with a single tagged subunit remains intact in the yeast extract [5], [13], [14], [36], [51]. Both FL-Ufo1 and the Ufo1-WD40 domain on beads supported the formation of SCF^Ufo1^-Ho-19S RP complexes and interacted with yeast ^myc^Cdc53, ^GFP^Ho, and with the tagged 19S RP complex. In addition endogenous Ddi1 was present as a major component of the ^GST^FL-Ufo1 and the ^GST^Ufo1-WD40 domain bead fractions of complexes formed in the presence of Ho. In the absence of Ho, we found an interaction of ^GST^Ufo1 with ^myc^Cdc53, but there was no interaction with Rpn11^GFP^. Ddi1 could still be detected in the ^GST^FL-Ufo1 and the ^GST^Ufo1-WD40 domain bead fractions, although in a considerably diminished amount (Figure 2A). A similar result was observed using tagged Rpn1^GFP^ (Figure S2). Rpn12 was present in the bead fraction indicating that 19S RP complexes and not just the tagged subunit were interacting with SCF^Ufo1^ (Figure S3).

Ddi1 is involved in the final stages of transfer of Ho and of Ufo1 to the 19S RP and could be recruited to the SCF^Ufo1^-Ho-19S RP complex after its assembly. We therefore repeated the above experiment using extracts of transformed *ddi1*Δ mutants. As in w.t. cells, Ho was crucial for formation of complex between SCF^Ufo1^ and the19S RP, however, there was no requirement for Ddi1 for formation of the SCF^Ufo1^-Ho-19S RP complex (Figure 2B). These results taken together and supported by our *in vivo* data that show that both Ho and Ufo1 accumulate as ubiquitylated conjugates in *ddi1Δ* mutants suggest that *in vivo* Ddi1 is recruited to the SCF^Ufo1^-Ho-19S RP complex after its assembly.

### SCF^Ufo1^-Ho-Ddi1-19S RP Complexes can be Reconstituted *in vitro* with Immobilized ^GST^Ddi1 or ^GST^Rpn1

**Figure 3:**
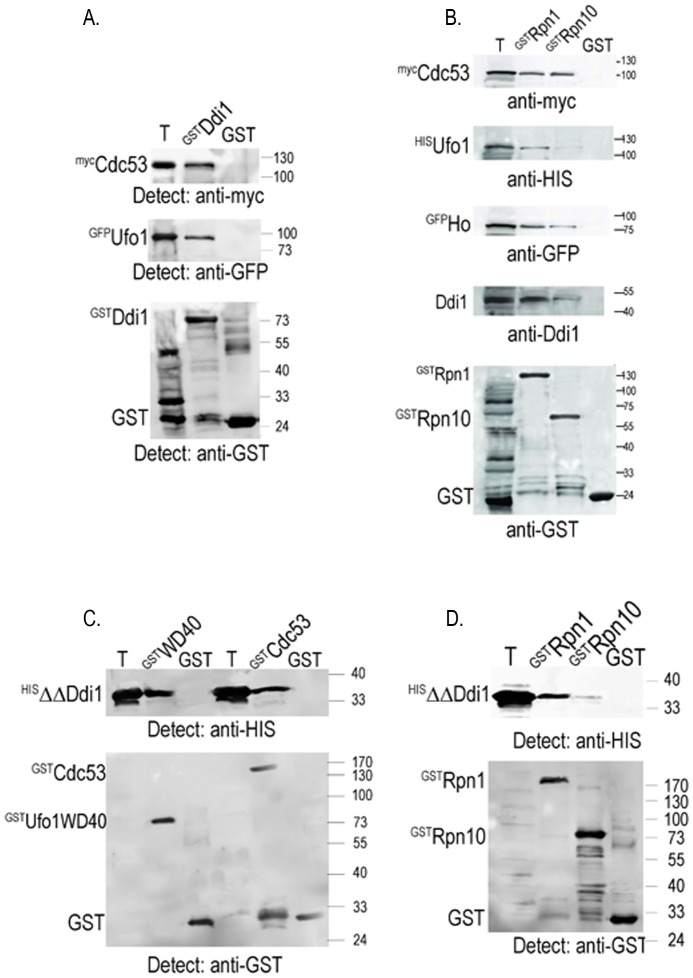
Immobilized Ddi1 and Rpn1 reconstitute SCF^Ufo1^ complexes *in vitro*. A. ^GST^Ddi1 or control GST on GSH beads were incubated with yeast extract from cells that produced ^myc^Cdc53 and ^GFP^Ufo1. Analysis was by Western blotting with anti-myc, anti-GFP, and anti-GST antibodies. T represents 10% of the yeast extract with which the beads were incubated. B.^ GST^Rpn1, ^GST^Rpn10, or GST beads were incubated with yeast extract from cells that produced ^myc^Cdc53 and ^GFP^Ho mixed with bacterial lysate with recombinant ^HIS^Ufo1. The bead fraction was analysed by Western blotting with anti-myc, anti-HIS, anti-GFP, anti-Ddi1, and anti-GST antibodies. T represents 10% of the yeast extract incubated with the beads. C. ^GST^Ufo1 WD40 domain, ^GST^Cdc53, or control GST on GSH beads were incubated with bacterial lysate from cells that produced recombinant ^HIS^ΔΔDdi1. The bead fraction was analysed by Western blotting with anti-HIS and with anti-GST antibodies as indicated. T is 10% of the ^HIS^ΔΔDdi1 bacterial lysate incubated with the beads. D. The ^HIS^ΔΔDdi1 bacterial lysate was incubated with ^GST^Rpn1, ^GST^Rpn10, or GST beads and analysed as above.

Reconstitution of SCF^Ufo1^-Ho-19S RP complexes *in vitro* in the above experiments was achieved with ^GST^FL-Ufo1 or the ^GST^Ufo1-WD40 domain on beads. To determine whether complex reconstitution is also possible with immobilized ^GST^Ddi1 or ^GST^Rpn1, the 19S RP subunit bound by Ddi1 [36], [52], we incubated ^GST^Ddi1 or control GST on GSH beads with yeast extract from cells that produced ^myc^Cdc53 and ^GFP^Ufo1. We observed a robust interaction of both proteins with ^GST^Ddi1 that was not observed with the GST control beads (Figure 3A). Similarly ^GST^Rpn1 on beads could reconstitute SCF^Ufo1^-^GFP^Ho-^GST^Rpn1 complexes that included endogenous Ddi1 present in the yeast extract (Figure 3B). No complexes were formed with the control GST beads. Thus it is possible to reconstitute complexes *in vitro* irrespective of which component is immobilized. Recombinant ^HIS^Ufo1 interacted extremely weakly with irrelevant control ^GST^Rpn10 beads, however we did observe an interaction of ^GFP^Ho and of ^myc^Cdc53 with this 19S RP subunit.

### The Core of Ddi1 Binds Cdc53, the Ufo1-WD40 Domain, and Rpn1

The Ufo1-UIMs fragment in isolation interacts with all three UbL-UbA proteins, Rad23, Dsk2, and Ddi1, however, FL-Ufo1 discriminates between them [35]. This suggests that the initial interaction between Ufo1 and Ddi1 occurs via interaction of its UIMs with the Ddi1-UbL domain and that specificity of UbL-UbA protein may be conferred by further interactions between Ufo1 and the core of Ddi1. We subcloned ^HIS^ΔΔDdi1 without the UbL and UbA domains comprising residues 180–325. Indeed both the ^GST^Ufo1-WD40 domain and ^GST^Cdc53 bound core ^HIS^ΔΔDdi1 (Figure 3C). Ddi1 binds the LRR domain of the Rpn1 subunit of the 19S RP [36], [52] via its UbL domain and here we found that the core ^HIS^ΔΔDdi1 fragment bound ^GST^Rpn1 robustly but showed only extremely weak binding to control ^GST^Rpn10 (Figure 3D). Thus after the initial interaction between the Ufo1-UIMs and the Ddi1-UbL these additional interactions with the Ddi1 core could secure Ddi1 within the SCF^Ufo1^-Ho-Ddi1-19S RP complex. They could also allow flexibility to the Ddi1-UbL allowing it to switch to binding Rpn1 for substrate or FBP transfer.

### SCF^Ufo1^-Ddi1-19S RP Complex Subunits Immunoprecipitate Together in the Presence of Ho

**Figure 4:**
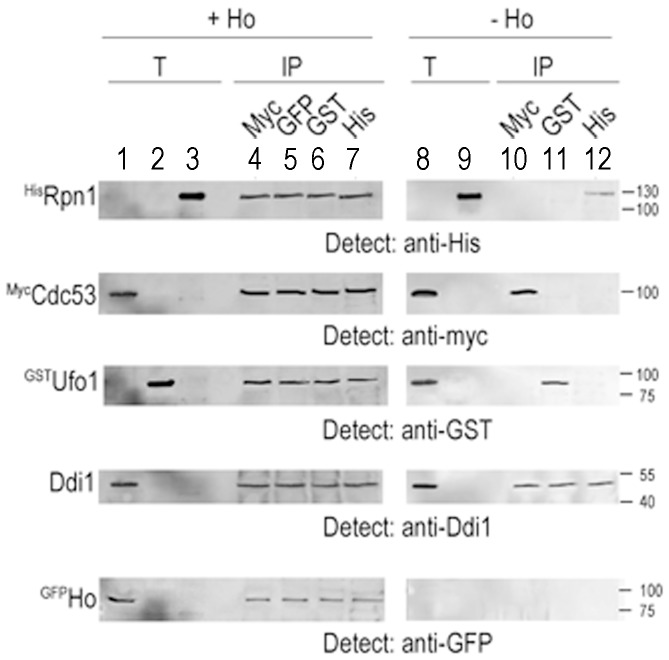
Cdc53, Ufo1, Rpn1 Ho and Ddi1 form a complex. Bacterial lysates from cells that produced ^HIS^Rpn1 or ^GST^Ufo1 were mixed with yeast extracts with ^myc^Cdc53 with or without ^GFP^Ho and divided into equal aliquots. Each aliquot was immunoprecipitated with a different antibody: anti-myc, anti-GFP, anti-GST or anti-HIS, in the presence of Protein A beads. The bead fractions were analysed by Western blotting with each antibody. **+ Ho**: Lanes 1–3: Lane 1. 10% of the total (T) extract from yeast that produced ^myc^Cdc53, ^GFP^Ho and endogenous Ddi1; Lane 2. Bacterial lysate with ^GST^Ufo1; Lane 3. ^HIS^Rpn1 bacterial lysate; Lane 4. anti-myc immunoprecipitation; Lane 5. anti-GFP immunoprecipitation; Lane 6. anti-GST immunoprecipitation; Lane 7. anti-HIS immunoprecipitation. **– Ho**: Lane 8. 10% of the yeast extract with ^myc^Cdc53 and endogenous Ddi1 and bacterial lysate with ^GST^Ufo1, Lane 9. 10% of bacterial lysate with ^HIS^Rpn1. Lane 10. anti-myc immunoprecipitation; Lane 11. anti-GST immunoprecipitation; Lane 12. anti-HIS immunoprecipitation;The IP lanes are headed by the antibodies used for immunoprecipitation of each protein.

The above experiments demonstrate that in the presence of Ho a SCF^Ufo1^-Ho-Ddi1-19S RP complex is formed *in vitro*. To verify that this is indeed a complex we prepared a reaction mix comprising yeast extract with ^myc^Cdc53, with or without ^GFP^Ho, and bacterial lysate with ^GST^Ufo1 and ^HIS^Rpn1, and immunoprecipitated each tagged protein separately. In the presence of Ho, immunoprecipitation of ^myc^Cdc53, of ^GFP^Ho, of ^GST^Ufo1 or of ^HIS^Rpn1 led to reciprocal coimmunoprecipitation of the other three proteins and of Ddi1 present in the yeast extract. In the absence of Ho, immunoprecipitation of ^myc^Cdc53, ^GST^Ufo1 or ^HIS^Rpn1 led to coimmunoprecipitation of endogenous Ddi1 from the yeast extract, but not of any of the other proteins of the complex formed in the presence of substrate. This result indicates that in the presence of Ho a bona fide complex is formed between SCF^Ufo1^-Ho-Ddi1 and Rpn1. This complex does not form in the absence of Ho (Figure 4).

### Ufo1 and Rpn1 Bind Ddi1 in Both a Competitive and a Synergistic Manner

#### (a) Competitive interaction: ^GST^Rpn1 abrogates binding of ^GFP^Ufo1 to ^HIS^Ddi1

**Figure 5:**
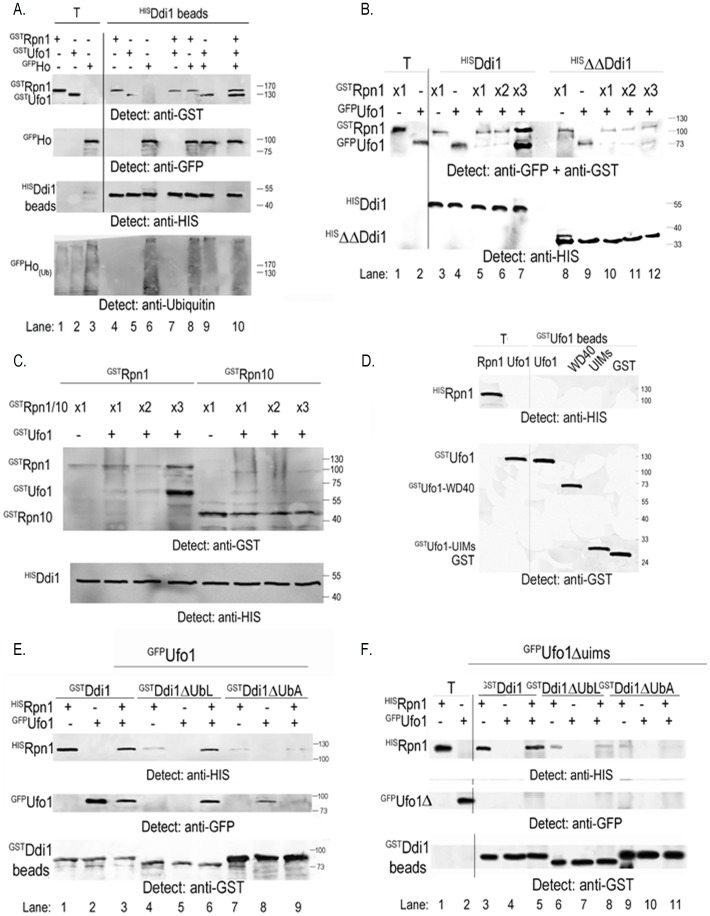
Rpn1 and Ufo1 exhibit synergistic binding to Ddi1. A. Bacterial lysate with ^GST^Rpn1 or ^GST^Ufo1 and yeast extract with ^GFP^Ho were incubated with ^HIS^Ddi1 beads alone (Lanes 4–6), in pairs Lanes 7–9), or all three together (Lane 10). The bead fractions were analysed by Western blotting with anti-GST, anti-GFP, anti-HIS and anti-ubiquitin antibodies. Lanes 1–3 (T) show 10% of the lysate/extract for bead incubation. B. Yeast extract with ^GFP^Ufo1 was incubated with ^HIS^Ddi1 and ^HIS^ΔΔDdi1 beads in the absence or the presence of increasing amounts of ^HIS^Rpn1. The bead fractions were analysed by Western blotting with anti-GFP, anti-GST antibodies and anti-HIS. Lanes 1 and 2 (T) show 10% of the lysate/extract with which the beads were incubated. C. Bacterial lysate with ^GST^Ufo1 was mixed with increasing amounts of lysate with ^GST^Rpn1 or ^GST^Rpn10 and incubated with nickel beads with ^HIS^Ddi1. The Western blots were analysed with anti-GST and anti-HIS antibodies. D. Bacterial lysate with ^HIS^Rpn1 was incubated with ^GST^Ufo1, the ^GST^Ufo1-WD40 domain, the ^GST^Ufo1-UIMs or control GST beads. The Western blots were analysed with anti-HIS and anti-GST antibodies. T indicates 10% of the lysate with which the beads were incubated. E. Recombinant ^HIS^Rpn1 made in bacteria and ^GFP^Ufo1 from yeast extract were incubated alone or together with GSH beads bound to ^GST^Ddi1, ^GST^Ddi1ΔUbL, or ^GST^Ddi1ΔUbA produced in bacteria. The bead fractions were analysed by Western blotting with anti-HIS and anti-GFP antibodies to show proteins that bound the GSH beads. The latter were detected with anti-GST antibodies. F. As above except that ^GFP^Ufo1Δuims was used instead of FL-Ufo1. T denotes 10% of the yeast extract incubated with the beads.

**Figure 6:**
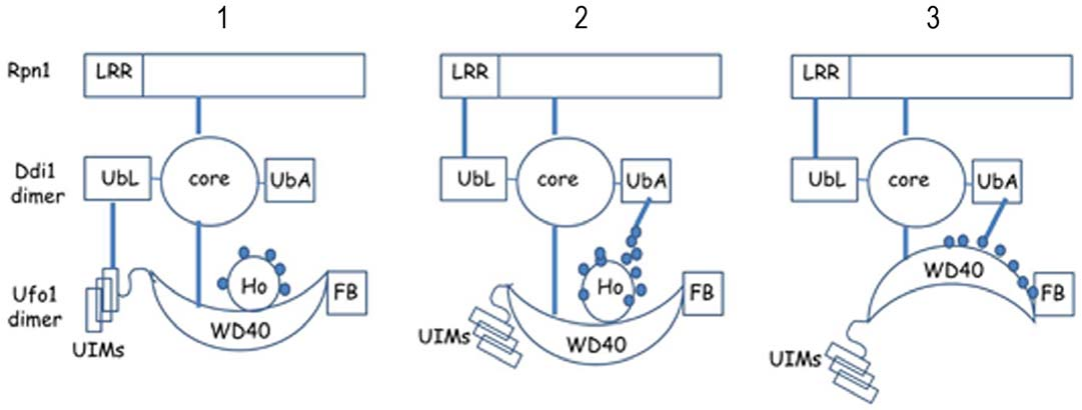
Model for sequential interactions of Ho, Ufo1, and Rpn1 with Ddi1. Panel 1. Active SCF^Ufo1^-Ho complexed with the 19S RP recruits Ddi1 by interaction of the Ufo1-UIMs with the Ddi1-UbL domain ([35] and Figures 2, 4 and 5F). Subsequently the core of Ddi1 binds the Ufo1-WD40 domain and Rpn1 (Figure 3C and D). Both Ufo1 (Figure 1) and Ddi1 [38] form dimers but are drawn here as monomers for clarity. Panel 2. The Ddi1-UbA domain interacts with ubiquitin chains on Ho and the Ddi1-UbL binds Rpn1 for transfer of ubiquitylated Ho to the 19S RP [34]. At this stage Ho, Ufo1, and Rpn1 bind Ddi1 simultaneously (Figure 5A). Panel 3. After degradation of Ho, Ufo1 can no longer bind Ddi1 in the presence of Rpn1 (competitive interaction, Figure 5B and C). However, at high levels of Rpn1 there is synergistic binding that is supported to a small extent by the Ddi1 core (Figure 5B) and is totally dependent on the Ddi1-UbA domain (Figure E). Based on the higher affinity of the Ddi1-UbL for Rpn1 seen in the competitive interaction we propose that at this stage the Ddi1-UbL binds Rpn1 and the Ddi1-UbA binds ubiquitin chains on Ufo1. This would lead to degradation of Ufo1 [36].

The Ddi1-UbL domain binds both the Ufo1-UIMs and Rpn1 [35], [52], however, interaction between Ddi1 and Rpn1 is essential for turnover of Ufo1 [36]. Both Ufo1 and Rpn1 bind the core of Ddi1 (Figure 3C and 3D) and this interaction may facilitate the switch of the Ddi1-UbL domain from the Ufo1-UIMs to Rpn1 for transfer of Ho or Ufo1 to the 19S RP. We therefore examined whether there is competition between Ufo1 and Rpn1 for interaction with Ddi1. Each protein incubated separately with Ddi1 beads was present in the ^HIS^Ddi1 bead fraction (Figure 5A, Lanes 4–6). However, Rpn1 displaced Ufo1 from Ddi1 when both ^GST^Ufo1 and ^GST^Rpn1 were incubated together with the ^HIS^Ddi1 beads (Lane 7). In contrast addition of yeast extract with ubiquitylated ^GFP^Ho to the reaction mix with either ^GST^Ufo1 or ^GST^Rpn1 did not affect the binding of either protein to ^HIS^Ddi1 (Lanes 8 and 9). Furthermore, Ho in the reaction mix comprising Ufo1, Rpn1, and Ddi1, abrogated the competition between Ufo1 and Rpn1 and all three proteins bound the ^HIS^Ddi1 beads (Lane 10) and Figure 2. Thus Ho protects Ufo1 from displacement from Ddi1 by Rpn1. In this complex the Ddi1-UbL would bind Rpn1, Ufo1 would be bound via its WD40 domain to Ho and to the Ddi1 core, and further interactions would occur between the Ddi1-UbA and the Ub chains on Ho. This is the complex we predict to underlie transfer of ubiquitylated Ho to the 19S RP (Figure 6).

#### (b) Synergistic interaction: ^GST^Rpn1 and ^GFP^Ufo1 bind ^HIS^Ddi1 in a tertiary complex that requires the Ddi1 UbA domain and does not involve the Ddi1 UbL domain

The competitive interaction between Ufo1 and Rpn1 may occur during handover of the FBP to the 19S RP after degradation of Ho. To explore this hypothesis we examined whether exclusion of ^GST^Ufo1 from binding to ^HIS^Ddi1 by ^GST^Rpn1 is concentration dependent. We calibrated the system by determining an amount for each lysate/extract that would give detectable binding of protein to the Ddi1 beads (x1, Figure 5B, Lanes 3 and 4). Then keeping the amount of ^GFP^Ufo1 extract constant in a fixed reaction volume we increased the amount of ^GST^Rpn1 lysate two- and threefold. In this experiment we used ubiquitylated ^GFP^Ufo1 produced in yeast [35]. ^GST^Rpn1 at x1 and x2 in the reaction mix gave a similar amount bound to the Ddi1 beads. Both these ^GST^Rpn1 concentrations abrogated binding of ^GFP^Ufo1 to Ddi1 (Figure 5B, Lanes 5 and 6 and as observed in Figure 5A, Lane 7). However, x3 the amount of ^GST^Rpn1 lysate induced synergistic binding of ^GST^Rpn1 and ^GFP^Ufo1 to the ^HIS^Ddi1 beads. A similar although considerably weaker signal was obtained when core ^HIS^ΔΔDdi1 beads were used. In contrast binding of ^GST^Rpn10 to the ^HIS^Ddi1 beads was not affected by ^GST^Ufo1 nor was any synergistic effect observed between them in binding to Ddi1 (Figure 5C). In contrast to Ddi1 [36] there is no direct binding between Ufo1 and Rpn1 (Figure 5D).

The competition between Ufo1 and Rpn1 for binding Ddi1 may involve the Ddi1-UbL which binds both proteins (above). To address this question we repeated the synergistic binding experiment described in Figure 5B but this time in addition to ^GST^FL-Ddi1 beads we used Ddi1 that lacked either the UbL or UbA domain: ^GST^Ddi1ΔUbL, and ^GST^Ddi1ΔUbA, respectively (Figure 5E, Lanes 1–3). Ddi1ΔUbL exhibited severely reduced binding to Rpn1 and did not bind Ufo1 when each protein was incubated separately with the beads. In contrast, Ddi1ΔUbL bound both Rpn1 and Ufo1 synergistically when both were present in the reaction mix. This suggests a role for the Ddi1-UbA in the synergistic binding of Rpn1 and Ufo1 to Ddi1. Surprisingly although Rpn1 binds the Ddi1-UbL, when we incubated ^HIS^Rpn1 with ^GST^Ddi1ΔUbA beads it interacted less strongly than with ^GST^FL-Ddi1 beads (Figure 5E, compare Lane 1 with Lane 4). ^GFP^Ufo1 bound ^GST^Ddi1ΔUbA beads and there was an extremely weak synergistic binding of Rpn1 and Ufo1 to ^GST^Ddi1ΔUbA beads when both were present in the reaction mix. Our previous *in vivo* experiments indicated that Ufo1 and Ddi1 interact via the Ufo1-UIMs and the Ddi1-UbL [35]. We therefore substituted ^GFP^Ufo1Δuims for ^GFP^FL-Ufo1. Indeed ^GFP^Ufo1Δuims did not interact with ^GST^FL-Ddi1, ^GST^Ddi1ΔUbL or ^GST^Ddi1ΔUbA beads both in the presence or the absence of Rpn1 (Figure 5F).

## Discussion

Complex reconstitution *in vitro* indicated that SCF^Ufo1^ complexes that contain their substrate, Ho, are associated with the 19S RP. These complexes can assemble in the absence of Ddi1, however, in experiments with extracts from w.t. cells Ddi1 is found in association with the SCF^Ufo1^-Ho-19S RP complex. Our interpretation is that Ddi1 is recruited to preformed SCF^Ufo1^-Ho-19S RP complex. Based on our previous experiments *in vivo* we propose that Ddi1 enters the SCF^Ufo1^-Ho-19S RP complex via initial interaction between the Ufo1-UIMs and the Ddi1-UbL ([35] and Figure 5F). Subsequent interaction between the Ufo1-WD40 and the core of Ddi1 detected here could explain the specificity of the interaction of SCF^Ufo1^ for Ddi1 [35]. The recruitment of Ddi1 after formation of the SCF^Ufo1^-Ho-19S RP complex supports our *in vivo* results that suggested Ddi1 is required for disassembly of SCF^Ufo1^ complexes after substrate degradation. This hypothesis is based on accumulation of ubiquitylated Ho in the cytoplasm of *ddi1Δ* mutants [34], stabilization of full-length Ufo1 in *ddi1Δ* mutants, cell cycle arrest at the G_1_-S interphase by overexpression of *UFO1Δuims* in wild type cells or of full-length *UFO1* in *ddi1Δ* mutants, and by the accumulation of Cln2, a substrate of the FBP, Grr1 [21], in cells with a high level of Ufo1Δuims [35].

The Ufo1-UIMs promote dimerization of Ufo1 and are crucial for all interactions of Ufo1 with Ddi1. They may fulfill two roles in dimerization: one is physical interaction between the UIMs of two Ufo1 molecules to initiate dimerization. The other is regulation of access to the Ufo1-WD40 domain as full-length Ufo1 did not dimerize with an Ufo1-WD40 domain fragment. Thus dimerization may start at the C-terminal UIMs and proceed to include the Ufo1-WD40 domains. We previously reported that SCF complexes from cells that produced Ufo1Δuims are capable of degrading Ho [35]. Given that dimerization of FBPs has been shown to be a prerequisite for substrate ubiquitylation in some instances [28], [32], [33], our current results support an interpretation that in the absence of its UIMs the Ufo1-WD40 domains of each monomer are able to interact with one another *in vivo*. The Ufo1-WD40 domain alone is sufficient for formation of complexes that include ^GFP^Ho, the 19S RP, and Ddi1 and indeed in our yeast two-hybrid experiments we reported an interaction between Cdc53 and the Ufo1-WD40 domain [35]. This is unusual as the solved SCF structures do not display interaction between the cullin and the WD40 domain of the FBP [23], [25] or with the related BTB/3-box domain receptor protein [28]. The Ufo1 WD40 sequence has a rather degenerate β-propeller sequence and a full analysis of this unusual interaction awaits solution of the 3D structure of Ufo1. A dimerization sequence has been identified in the N-terminal region of certain FBPs [26], [27] and it is conceivable that there is one in Ufo1 too that could serve for dimerization in the absence of the Ufo1 UIMs.

The Ddi1-UbL – Ufo1-UIMs interaction is essential for recruitment of Ddi1 to the SCF^Ufo1^-Ho-19S RP complex ([35] and Figure 5F). However, degradation of the ubiquitylated substrate requires transfer of the Ddi1-UbL from its interaction with the Ufo1-UIMs to Rpn1 [36]. In the presence of Ho a complex is formed that includes Ufo1, Rpn1, and Ddi1. Binding of Ho to Ddi1 is mediated by interaction of its ubiquitin chains with the Ddi1-UbA domain [34]. We propose that interaction of the Ddi1-UbA with a critical amount of Ub chains on Ho could lead to switching of the Ddi1-UbL domain from the Ufo1-UIMs to Rpn1 for transfer of Ho to the 19S RP. Transfer of the Ddi1-UbL without disruption of the complex between these proteins would be supported further by concurrent binding of the Ddi1 core to both Ufo1 and Rpn1 and by interactions of Ho with both the Ufo1-WD40 domain [40] and with the Ddi1-UbA domain via its Ub chains ([34] and Figure 6).

The “substrate shield” model proposes that the substrate protects the FBP from degradation [47]. In the reaction lacking Ho (comparable to an *in vivo* situation after substrate degradation but prior to SCF^Ufo1^ complex disassembly) we observed two different modes of interaction of Ufo1 and Rpn1 with Ddi1: (a) competitive - Rpn1 excludes Ufo1 from binding to Ddi1; (b) synergistic - high levels of Rpn1 formed a tertiary complex between Ufo1, Ddi1, and Rpn1. The competitive interaction indicates that the Ddi1-UbL has higher affinity for Rpn1 than for the Ufo1-UIMs. The dependence of synergistic binding of Ufo1 and Rpn1 on the Ddi1-UbA domain suggests that Ub chains on Ufo1 are involved. The higher ratio of Rpn1 to Ufo1 in the *in vitro* Ddi1 synergistic binding experiment could parallel molecular crowding within the SCF^Ufo1^-19S RP complex. Thus in the absence of Ho our data support a complex in which the Ddi1-UbL is bound to Rpn1 while the Ddi1-UbA domain binds Ub chains on Ufo1. This model for sequential transfer of Ho and of Ufo1 to the 19S RP is presented in Figure 6.

## Materials and Methods

### Yeast Strains

Wild type BY4741 (*MAT* ***a*** *; his3Δ1; leu2Δ0; met15 Δ0; ura3Δ0*), and *ddi1Δ (MATα, his3 Δ1, leu2 Δ0, lys2 Δ0, ura3 Δ00, YER143w::kanMX4)* were purchased from Euroscarf. Strains with genomic *RPN1-GFP* and *RPN11-GFP* are from the library of [48].

### Bacterial Strains

Rosetta bacteria (Novagen) (F–, *omp*T, *hsd*S_B_(r_B_-, m_B_-), *dcm*, gal, *lacY1,* pRARE (*argU, argW, ileX, glyT, leuW, proL*) (Cm^R^) were used for most recombinant protein expression. His-tagged recombinant proteins were expressed in BL21 (Promega) (F–, *omp*T, *hsd*S_B_(r_B_-, m_B_-), *dcm*, *gal,* λ(DE3), pLysS (Cm^r^)) or M15 (Qiagen) (*NaI^S^, str^S^, rif^S^, thi^-^, lac^-^, ara^+^, gal^+^, mtl^-^,F^-^, recA^+^, uvr^+^, lon^+^(Km^r^*)) bacteria.XL1 MRF1 (StrataGene) (*Nalr) gyrA96 end A1 Δβ(lacz)M15/recA1 laclq proA+B+ lacF’::Tn10 relA1 supE44 thi hsdR17(rk-mk*+) was used for plasmid amplification.

### Yeast Plasmids

*YCPGAL-GFP* (*GFP-UFO1, GFP- ΔUIMs, GFP-HO)* are described in [35]; p*MT2989* for expression of *MYC-CDC53* from the *GAL* promoter was obtained from M. Tyers [21]. p*YE-RPN11*-*GFP* in which expression of *RPN11-GFP* is from the *ADH1* promoter was obtained from M. Glickman [49].

**Growth media and yeast transformation** by LiOAc are as in [50].

### Bacterial Plasmids

p*HB2-GST-CDC53,* p*GST-DDI1,* p*GST-DDI1-ΔUBL,* p*GST-DDI1-ΔUBA,* p*HIS-DDI1,* p*HIS-ΔΔDDI1*, p*GST-RPN1*, and p*GST-RPN10* (gift of Dorota Skowyra); p*GEX-5X-1* (Amersham Biosciences) was used to construct p*GST-UFO1* by amplifying the *UFO1* gene from genomic DNA using primer pair Ufo1F (GAATTCATGGAGCGGCCTGGCTTGGTATT) and Ufo1R (CTCGAGTCAATTGATTTCACTCAATGACAACG). pGST-UIMs was constructed using Ufo1UIMsF **(** GAATTCAAAACGACATTCAGTTGAGAATTGCA **)** and Ufo1R. pGST-WD40 employed primer pair WD40GstF (GAATTCAATATTAATGCTGCAGTG) and WD40GstR (CTCGAGGTTTTCTTCATCGGTGTC). p*CDFduet1- HIS-UFO1* was constructed by amplifying the *UFO1* ORF with primer pair Ufo1HisF (GGATTCATGG AGCGGCCTGGCTTGGTATT) and Ufo1HisR (CTCGAGTCAATTGATTTCACTCAATG ACAACG). p*CDFduet1- HIS-WD40* was constructed by amplifying the WD40 domain of *UFO1* with primer pair WD40HisF (GGCGCGCCAATATTAATGCTGCAGTG) and WD40HisR (CTCGAGGTTTTCTTCATCGGTGTC).

**Immunoprecipitation and immunoblotting** were performed as described in [5], [40]. Briefly, proteins were induced from the *GAL* promoter by overnight growth in minimal medium with 2% galactose. Next morning the culture was diluted 1∶3 and grown for a further 1.5 hours. 50 mls of logarithmic culture served as the source of a 300 µl extract with 80 µg/µl protein. 200 µl were taken for immunoprecipitation (IP) with the appropriate antibody and the immunoprecipitate was run in a single lane for Western blotting (WB).

### Antibodies

Mouse anti-GFP (Roche Applied Science), mouse 9E10 anti-myc (Enzo), and mouse anti-HIS (Sigma) antibodies were used at a dilution of 1∶250 for IP and at 1∶1,000 for WB; mouse anti-GST (Santa Cruz Biotechnology) antibodies were diluted 1∶1,000 for IP and 1∶2,000 for WB, rabbit anti-Ddi1 (gift from Jeffrey Gerst) was used at 1∶5,000 for WB. Goat anti-mouse and anti-rabbit antisera, used at 1∶1,000 were from Santa Cruz Biotechnology. Protein A-sepharose was purchased from Amersham and used at 50%; 30 µl were added to each sample.

**TCA precipitation** proteins were precipitated from 300 µl cell extract by adding TCA to 10% with 10 minutes incubation on ice. The pellet was centrifuged at 12,000 g for 10 minutes and five volumes of cold acetone were added. The protein pellets were harvested and dried. For WB analysis the pellets were dissolved in 30 µl of sample buffer and 5 µl of each fraction was separated by SDS-PAGE.

### Expression of GST and HIS Fusion Proteins in Rosetta Bacteria

Bacteria were transformed by electroporation and the colonies selected on LB-agar plates with ampicillin and kanamycin, each at 100 µg/ml, and chloramphenicol at 34****µg/ml. A single colony was grown in 1 liter of LB (with ampicillin and chloramphenicol) to an OD_600_ of 0.6–0.8 (3–5 hours) with vigorous agitation at 37°C. IPTG was added to 0.4 mM to induce expression and the culture was incubated overnight at 20°C. The cells were harvested by centrifugation at 4°C for 10 min at 6,000 rpm. The cell pellet was washed with 20 ml of ice-cold PBS and resuspended in 3 ml yeast extract buffer (50 mM Tris-Cl pH 7.5, 150 mM NaCl, 5 mM EDTA, 0.1% NP40, 1∶25 Protease Inhibitor cocktail (Roche)). The cell suspension was disrupted with an ultra-sound sonicator on ice using 6 cycles each of 10 seconds and clarified by centrifugation for 10 min at 4,000 rpm at 4°C. The supernatants with the GST-fusion proteins were incubated with Glutathione-sepharose 4B (GSH) beads (Amersham Biosciences) prewashed in yeast extract buffer with 1% Triton-X100**;** HIS-fusion proteins were incubated with washed Ni-sepharose (Clontech) for 1.5 hr at 4°C. The bead fractions were washed 5 times in extract buffer with 2.5% Triton-X100. The GST- and HIS-fusion proteins on beads were stored at -20°C after addition of glycerol to 5%.

### GST *in vitro* Binding Assay

Yeast cells were grown overnight to late log phase (OD_600_ = 0.8) in 2% galactose medium for the *GAL*-regulated constructs, or in YePD. The cells were harvested by centrifugation at room temperature for 5 minutes at 4,000 rpm, washed in 50 ml TE and resuspended in 600 µl extract buffer. 0.5–0.6 mg of glass beads were added and the cells were broken by vigorous vortexing for 25 minutes at 4°C. The extract was clarified by centrifugation at 12,000 g for 20 minutes at 4°C and protein concentration was measured with the Bio-Rad protein reagent. 5–10 mg of protein extract were taken for each GST pull-down in a total volume of 350–400 µl extract buffer. 30–50 µl of 50% Glutathione Sepharose 4B beads coupled to GST fusion protein were added to each sample and incubated at 4°C for 1–2 hours with very mild shaking. The samples were washed 6 times with extract buffer with 2.5% Triton X100. The pellet was resuspended in 30–50 µl sample buffer x2, boiled for 5 minutes and centrifuged for 3 minutes at high speed to remove insoluble material. The supernatant was separated on a 12% polyacrylamide SDS gel with protein size standards followed by WB analysis.

### HIS-tagged Protein *in vitro* Binding Assay

As above, but with 30–50 µl of 50% Ni Sepharose beads coupled to the HIS fusion protein added to each sample and incubated at 4°C for 1–2 hours with very mild shaking. The samples were washed 6 times with extract buffer with 2.5% Triton X100 and 100 mM Imidazole. The pellet was resuspended in 30–50 µl sample buffer x2, boiled for 5 minutes and centrifuged for 3 minutes at high speed to remove insoluble material. The supernatant was separated on a 12% polyacrylamide SDS gel with protein size standards followed by WB analysis.

## Supporting Information

Figure S1 **Domains of Ufo1 and Ddi1 used in experiments.** The protein fragments used in the experiments depicted in the Figures are shown.
(TIF)

Figure S2 **Formation of SCF^Ufo1^-Ho-19S RP complex with yeast extract from** ***RPN1-GFP*** **cells.** ^GST^Ufo1 or control GST beads were incubated with yeast extract from cells with tagged genomic *RPN1-GFP* that were cotransformed with p*GFP-HO* and with p*MYC-CDC53.* The bead fraction was analysed by Western blotting with anti-GFP antibodies to detect Rpn1 and Ho, with anti-myc antibodies to detect Cdc53, and with anti-Ddi1 antibodies.
(TIF)

Figure S3 **Rpn12 is present in the ^GST^Ufo1 bead fraction.** A further experiment in which ^GST^Ufo1 and GST beads were incubated with yeast extract in the presence of ^GFP^Ho as in Figures 2 and S2. Here the Western blot employed antibodies made to ^GST^Rpn12. The presence of Rpn12 is an indication that the 19S RP is intact.
(TIF)
