# Supplementary figures and images for: Transfer of Ho Endonuclease and Ufo1 to the Proteasome by the UbL-UbA Shuttle Protein, Ddi1, Analysed by Complex Formation In Vitro
Source: PLoS One. 2012 Jul 16;7(7):e39210. doi: 10.1371/journal.pone.0039210 (PMC3398040; doi:10.1371/journal.pone.0039210)

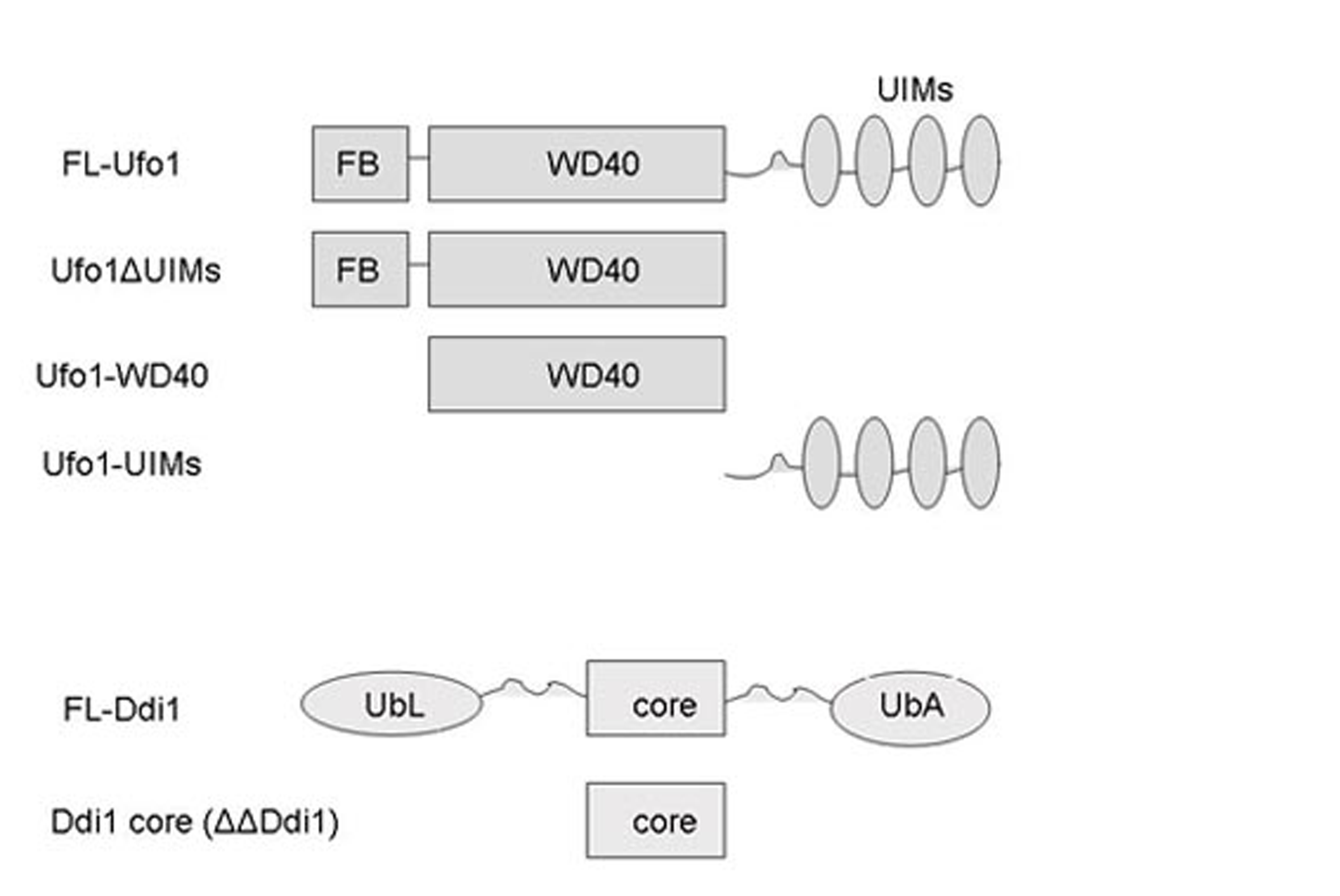

Supplement: Figure S1 — Domains of Ufo1 and Ddi1 used in experiments. The protein fragments used in the experiments depicted in the Figures are shown. (TIF) [file pone.0039210.s001.tif]

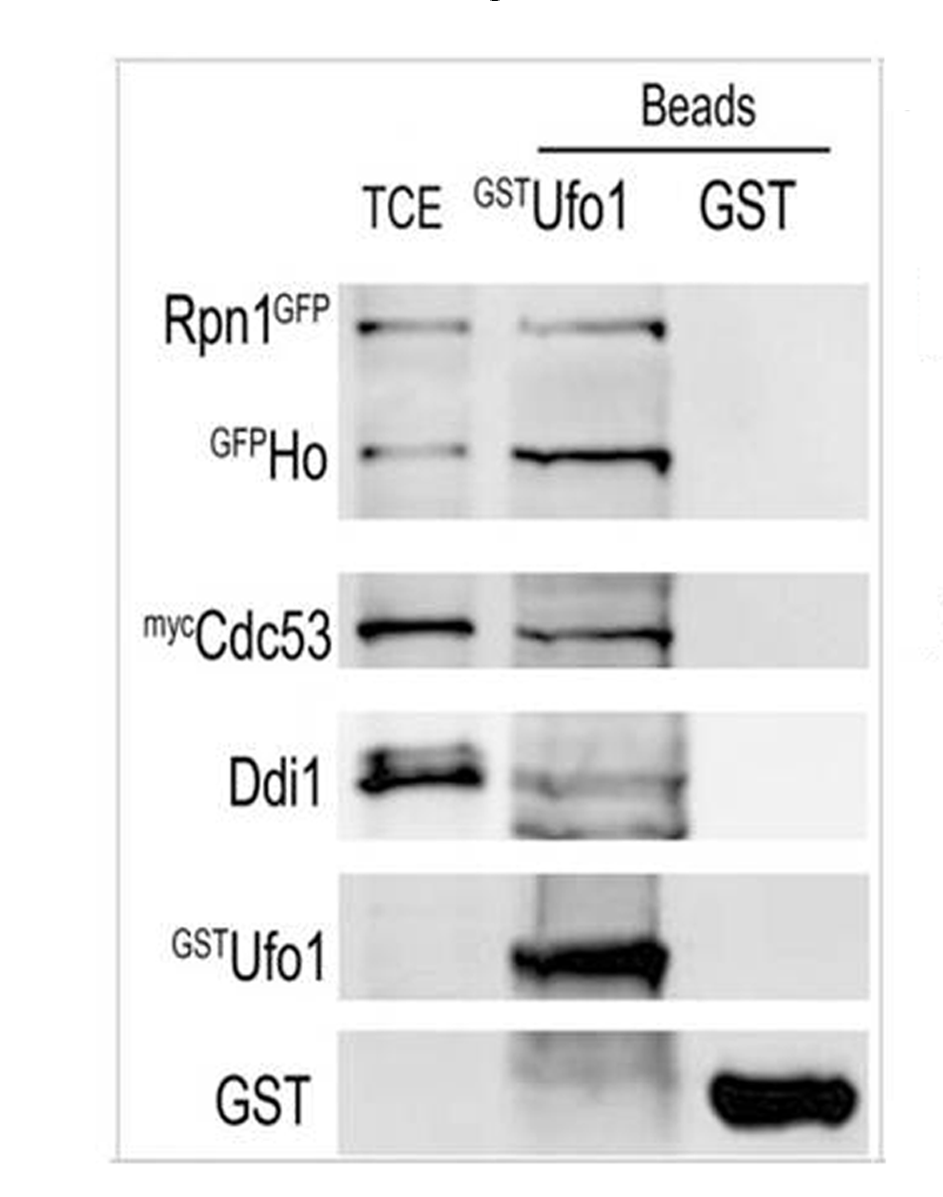

Supplement: Figure S2 — Formation of SCFUfo1-Ho-19S RP complex with yeast extract from RPN1-GFP cells. GSTUfo1 or control GST beads were incubated with yeast extract from cells with tagged genomic RPN1-GFP that were cotransformed with pGFP-HO and with pMYC-CDC53. The bead fraction was analysed by Western blotting with anti-GFP antibodies to detect Rpn1 and Ho, with anti-myc antibodies to detect Cdc53, and with anti-Ddi1 antibodies. (TIF) [file pone.0039210.s002.tif]

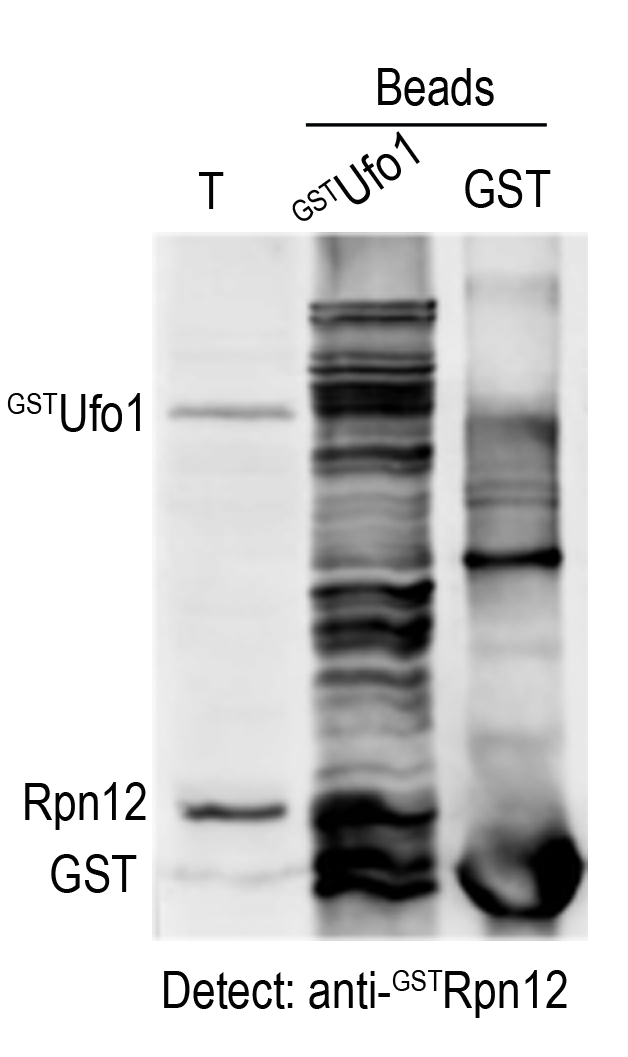

Supplement: Figure S3 — Rpn12 is present in the GSTUfo1 bead fraction. A further experiment in which GSTUfo1 and GST beads were incubated with yeast extract in the presence of GFPHo as in Figures 2 and S2. Here the Western blot employed antibodies made to GSTRpn12. The presence of Rpn12 is an indication that the 19S RP is intact. (TIF) [file pone.0039210.s003.tif]
